# PRO-DIALOG—the effect of a novel dialogue-based parent-teacher conference on mental health in kindergarten children: a cluster randomized controlled trial

**DOI:** 10.1186/s13063-025-08980-x

**Published:** 2025-08-21

**Authors:** Ingvar Bjelland, Gro Janne Wergeland, Christopher Gillberg, Rolf Gjestad, Adrijana Višnjić-Jevtić, Veronica Kibbe Lisæth, Carmela Miniscalco, Amanda Louise Flygel Tufta, Ida Lygre Vermeer, Alicja Renata Sadownik, Philip Wilson, Maj-Britt Posserud

**Affiliations:** 1 Department of Child and Adolescent Mental Health Services, Division of Psychiatry, Haukeland University Hospital, Bergen, Norway; 2 Department of Clinical Medicine, University of Bergen, Bergen, Norway; 3 Gillberg Neuropsychiatry Centre, Institute of Neuroscience and Physiology, University of Gothenburg, Gothenburg, Sweden; 4 Centre for Crisis Psychology, University of Bergen, Bergen, Norway; 5 Faculty of Teacher Education, University of Zagreb, Zagreb, Croatia; 6 Child and Adolescent Neuropsychiatry Unit, Queen Silvia Children’s Hospital, Sahlgrenska University Hospital, Gothenburg, Sweden; 7 Department of Pediatrics, Haukeland University Hospital, Bergen, Norway; 8 User Representative, Bergen, Norway; 9 Kindergarten Knowledge Centre for Systemic Research On Diversity and Sustainable Futures (KINDknow), Western Norway University of Applied Science, Bergen, Norway; 10 University of Aberdeen, Aberdeen, UK; 11 Department of General Practice, University of Copenhagen, Copenhagen, Denmark

**Keywords:** Mental health, Health promotion, Secondary prevention, Screening, Shared decision making, Children, Kindergarten, Early detection, Early intervention, Preschool education

## Abstract

**Background:**

Mental health difficulties in preschool children often go unnoticed and may result in delayed access to potentially beneficial services. In Norwegian kindergartens, teachers get to know the children well over time and have parent-teacher conferences once or twice a year. Kindergarten is thus a well-suited arena for early identification and intervention of mental health difficulties. To address the need for a kindergarten-specific method for secondary prevention, we have developed Dialogue-Based Early Detection (DBED) in close collaboration with educators and parents in eight different kindergartens. Results from a feasibility study indicate that DBED works well as a parent-teacher collaborative screening method and is well accepted by the users. In the PRO-DIALOG project, we will examine the potential of DBED and explore its possible long-term effects in a randomized controlled trial.

**Methods:**

Ten kindergartens will be randomly selected to implement DBED, while ten will act as controls, offering ordinary parent-teacher conferences. Parents of at least 100 + 100 children will be recruited. The primary outcome will be children’s mental health in the intervention group as assessed by the Strengths and Difficulties Questionnaire (SDQ), during the 5-year follow-up, compared to the control group. Secondary outcomes will be (i) the effect of DBED on parental stress as measured by the Parental Stress Scale (PSS) and (ii) time to activation of support, as measured by the duration from the first parent-teacher conference to activation of any support, comparing the two groups. We will also assess (iii) socio-demographic predictors for mental health development, parent and teacher concern for the child, parental stress, and parent satisfaction with DBED, (iv) screening properties of DBED compared to SDQ, and (v) social validity of DBED as measured by user-satisfaction questionnaires and interviews with both parents and teachers.

**Discussion:**

This complex intervention study includes a wide range of outcomes beyond the mental health scores of kindergarten children. If the intervention is well accepted and has a positive influence on the children’s mental health, the DBED method has a potential for a wide dissemination. This study will produce new knowledge on kindergarten as an arena for the promotion of mental health among young children.

**Trial registration:**

ClinicalTrials.gov NCT06471816. Registered on 2024–06-22 16:06. Retrospectively registered.

**Supplementary Information:**

The online version contains supplementary material available at 10.1186/s13063-025-08980-x.

## Administrative information

Note: the numbers in curly brackets in this protocol refer to SPIRIT checklist item numbers. The order of the items has been modified to group similar items (see http://www.equator-network.org/reporting-guidelines/spirit-2013-statement-defining-standard-protocol-items-for-clinical-trials/)

**Table:** 

Title {1}	PRO-DIALOG – The effect of a novel dialogue-based parent-teacher conference on mental health in kindergarten children- a cluster randomized controlled trial.
Trial registration {2a and 2b}.	ClinicalTrials.gov Identifier: NCT06471816
Protocol version {3}	December 2024; third version.
Funding {4}	The Research Council of Norway, project number 344516
Author details {5a}	Ingvar Bjelland ^1, 2^ Gro Janne Wergeland ^1, 2^ Christopher Gillberg ^3^ Rolf Gjestad ^1, 4^ Adrijana Višnjić-Jevtić ^5^ Veronica Kibbe Lisæth ^1, 2^ Carmela Miniscalco ^3, 6^ Amanda Louise Flygel Tufta ^7^ Ida Lygre Vermeer ^8^ Alicja Renata Sadownik ^9^ Philip Wilson ^10, 11^ Maj-Britt Posserud ^1, 2, 3^ 1 Department of child and adolescent mental health services, Division of Psychiatry, Haukeland University Hospital, Bergen, Norway 2 Department of Clinical Medicine, University of Bergen, Bergen, Norway 3 Gillberg Neuropsychiatry Centre, Institute of Neuroscience and Physiology, University of Gothenburg, Sweden 4 Centre for Crisis Psychology, University of Bergen, Bergen, Norway 5 Faculty of Teacher Education, University of Zagreb, Croatia 6 Child and Adolescent Neuropsychiatry Unit, Queen Silvia Children’s Hospital, Sahlgrenska University Hospital, Gothenburg, Sweden 7 Department of Pediatrics, Haukeland University Hospital, Bergen, Norway 8 User representative, Bergen, Norway 9 Kindergarten Knowledge Centre for Systemic Research on Diversity and Sustainable Futures (KINDknow),Western Norway University of Applied Science, Norway 10 University of Aberdeen, United Kingdom 11 Department of General Practice, University of Copenhagen, Denmark
Name and contact information for the trial sponsor {5b}	Haukeland University Hospital, Randi-Luise Møgster, CEO of Division of Psychiatry, randi-luise.mogster@helse-bergen.no
Role of sponsor {5c}	Role of study sponsor: Responsible for data protection and personal protection. Role of funders: Controlling that the grant is used according to the intended purpose.

## Introduction

### Background and rationale {6a}

Childhood mental health has been proposed to be the strongest predictor of adult well-being [1]. The major costs of common mental health problems among children both for the individual and society are well known, and there are negative effects on mental and physical morbidity, alcohol and substance abuse, crime, relational problems, injuries, suicide, social, peer, and school-related problems, and sick leave [2, 3]. Early detection and intervention are therefore likely to be important to prevent secondary consequences of mental health problems, and early intervention may reduce socio-economic costs in the long run [4].

Ideally, all children with significant emotional, behavioral, or cognitive problems should be detected and offered adequate developmental support tailored to individual needs. Such support could be provided by community-level agencies or by child and adolescent mental health services (CAMHS), depending on the need. The help should be given as early as possible to support positive development, promote well-being, and achieve optimal educational and social gain.

Nevertheless, only a minority of children at risk is detected and offered appropriate interventions, younger children less often than older ones [5–8]. In Norway, most children (> 95%) attend a kindergarten from the age of 1 year. Law regulates the educational standards in the kindergartens, including requirements for staff and educational competence. Consequently, kindergartens are well suited for first-line detection and intervention for better mental health for children in general [9]. However, established screening programs for behavioral or emotional problems among children have been criticized by some users, i.e., kindergarten teachers and the parents of the children, for pathologizing natural variation in behavior and emotional reactions and for generating unnecessary concern for healthy children.

In the absence of a generally accepted screening tool adapted to ordinary kindergartens, we developed the Dialogue Based Early Detection (DBED) [10] as a specific mental health intervention for early years. After having achieved good feasibility data, we will now test its effectiveness in an RCT.

### Objectives {7}

The primary objective is to evaluate DBED’s impact on mental health for children in kindergarten measured by the Strength and Difficulties Questionnaire (SDQ) scores. However, for a better understanding of potential mechanisms, secondary objectives are to evaluate how DBED influences parental stress and activation of interventions for mental health concerns, to establish the screening properties of DBED to detect mental health problems among children in kindergarten, and evaluate its acceptability for kindergarten teachers.

### Trial design {8}

The study design is a cluster randomised open trial, where 20 kindergartens will be randomised to either DBED intervention (*n* = 10) or control groups (*n* = 10). In the control group, parent-teacher appraisals are performed in the traditional way (treatment as usual; TAU). Children’s mental health, as measured by SDQ scores, will be assessed by both parent- and teacher-report biannually through the last 3 years in kindergarten and for the first 2 years in school. Our analyses will examine DBED’s possible superiority to TAU. Similar analyses will be performed as to DBED’s impact on activation of interventions for mental health concerns and parental stress.

The number of components involved, the wide variety of behaviours targeted, expertise and skills required by those delivering the intervention, and the permitted level of flexibility of the intervention confirm that the PRO-DIALOG project is complex intervention research [11]. Hence, in addition to the core trial design, we will use a mixed-method approach to the process evaluation, including both quantitative and qualitative methods. A logic model of the project can be found in Additional file 1.

## Methods: participants, interventions and outcomes

### Study setting {9}

The participating kindergartens are all localized in Bergen or Øygarden municipalities, Norway, with approx. 290,000 and 40,000 inhabitants, respectively. These municipalities cover a mix of urban and sub-urban areas with no generally underprivileged localities. The kindergartens are managed by Haukeland University Hospital, Eventus barnehage AS, or Øygarden municipality.

### Eligibility criteria {10}

Inclusion criteria: Kindergartens owned by either Haukeland University Hospital, Eventus Barnehage AS, or Øygarden municipality, each of which offer regular parent-teacher conferences. Parents of children who are beginning their third last year of kindergarten attendance. This corresponds to children born 3 years prior to the inclusion year (i.e., children born in 2021 will be recruited in the autumn of 2024).

Exclusion criteria: Kindergarten manager rejecting invitation to participate. Parents who do not understand either Norwegian or English.

### Who will take informed consent? {26a}

Study coordinator will obtain informed consent digitally from trial participants through EasyTrial. When the consent is distributed to the participants, they will have to read through the document before they choose to sign or decline. To ensure that the correct person gives consent, each participant must sign with an eSigning solution connected to their social security number. Participants may withdraw their consent whenever they wish and without explanation.

### Additional consent provisions for collection and use of participant data and biological specimens {26b}

N/a.

## Interventions

### Explanation for the choice of comparators {6b}

DBED is a novel approach to the mandatory, though loosely defined, parent-teacher conference in kindergartens. Hence, the control condition (comparator) will be a traditional parent-teacher conference (“treatment as usual”). In a qualitative part of the PRO-DIALOG project, sound recording of parent-teacher conferences from both intervention and control kindergartens will be made to analyze and compare content and communication patterns.

### Intervention description {11a}

#### Intervention

**Fig. 1:**
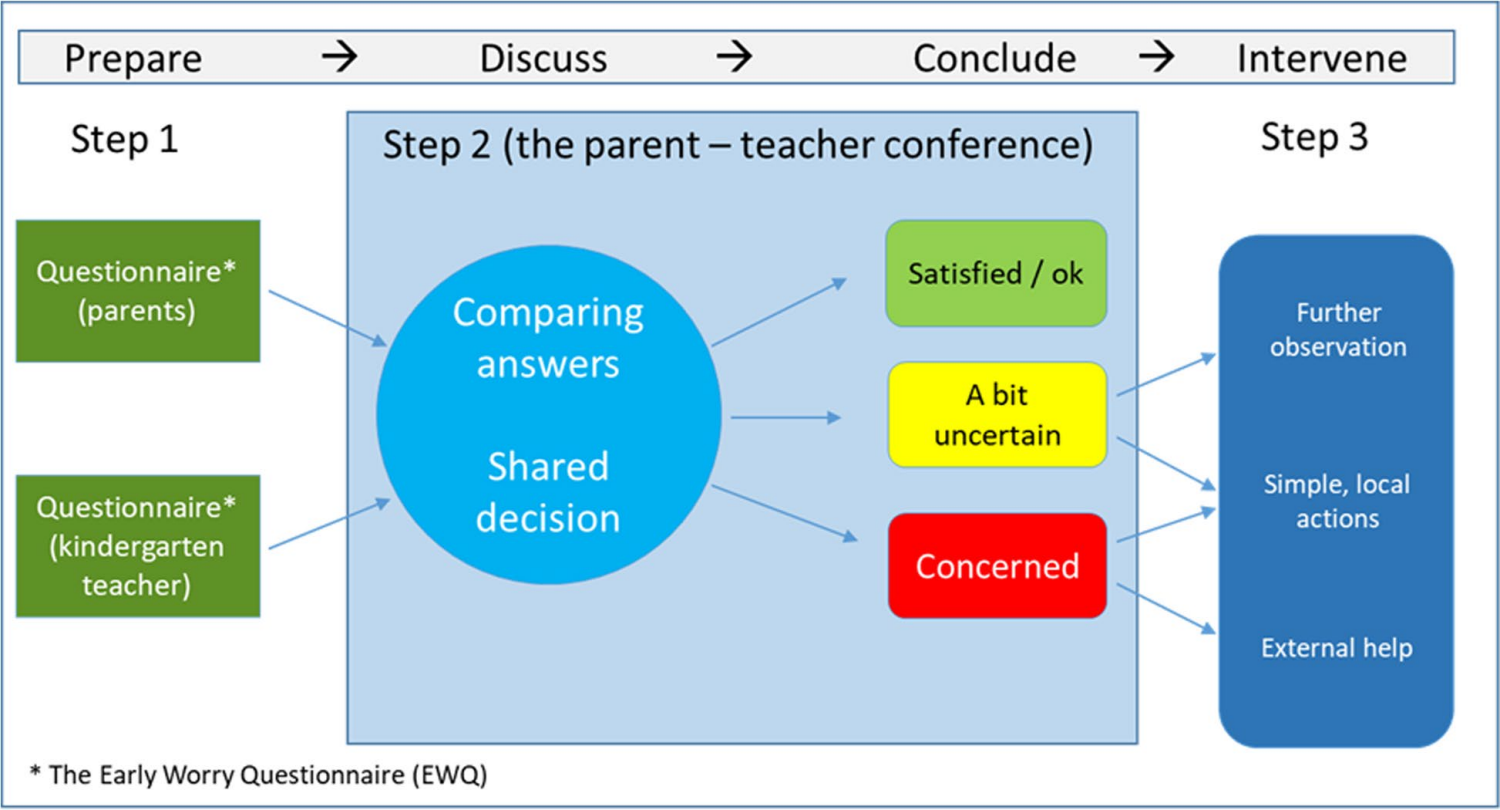
The steps of dialogue-based early detection

DBED [10] is a 3-step method (Fig. 1) starting with both parents and kindergarten teachers completing the Early Worry Questionnaire (EWQ) (Step 1) as a preparation for the biannual parent-teacher conference. The EWQ contains 37 questions that systematically map possible concerns for the individual child’s wellbeing and development. When a concern is stated, the respondents are asked to suggest possible causes in an open-ended follow-up question. Step 2 is the parent-teacher conference where they compare their answers from the EWQ and discuss whether there is a reason for concern or not. In case of consensus of concern, a discussion of possible further actions should follow (Step 3), such as closer observation of the child, simple educational interventions (e.g., playgroup), or referral to more specific external assessment or services. During feasibility testing (*N* = 153) of the method, DBED was well accepted by parents and teachers [10]. Preparation before parent-teacher conferences by completing the EWQ facilitated a more active involvement of the parents in the dialogue. They also more often presented previously unspoken concerns for their child [10].

### Criteria for discontinuing or modifying allocated interventions {11b}

The intervention is not designed to be modified or personally tailored outside the decision-making process at step 2.

### Strategies to improve adherence to interventions {11c}

The DBED is a semi-structured procedure for the parent-teacher conference. Kindergarten teachers and parents have been strongly involved in the development of DBED to achieve a user-friendly method. Managers of each kindergarten are accountable for adherence to the method. The project group has provided oral and written information about the purpose and procedures of the project to both teachers and parents. Every teacher has been equipped with their own tablet for completing questionnaires and communication with the project coordinator. The online digital questionnaires have been piloted for user optimization ahead of full-scale implementation of the project. After each half-year round of parent-teacher conferences and questionnaire completion, the project group arranges digital meetings with the teachers for feedback on their experiences. The project coordinator responsible for issuing and receiving the questionnaires will remind the separate kindergarten teacher when questionnaires are not returned in time. The completeness of the submitted EWQs and conclusions from the parent-teacher conference will indicate adherence.

### Relevant concomitant care permitted or prohibited during the trial {11d}

The intervention kindergartens should not perform traditional parent-teacher conferences in addition to the DBED for the participating parents.

### Provisions for post-trial care {30}

N/a: Due to the nature of the intervention (parent-teacher conference), adverse effects from trial participation are considered unlikely. The possible actions concluded during the DBED are all part of the standard supportive measures and care and identical to those initiated in the control kindergartens (and all other kindergartens in the same municipality).

### Outcomes {12}

Primary outcome:

- Effect of DBED on mental health measured as the difference between intervention group and control group in Total difficulties score of SDQ. Measurement scores will be recorded every sixth month during the entire 5-year project period (i.e., child-age 3 to 8 years) as repeated measures. SDQ sub-scores and two impact scores (difficulties in general and impact on four areas of daily functioning) will be analyzed. The primary endpoint will be during the last 3 months in kindergarten before the child starts school, giving total change over six time points of measurements. The secondary endpoint will be during the last 3 months in the second school year (10 time points of measurements).

Secondary outcomes:

- Effect of DBED on time to initiated locally available supportive action analyzed as the difference in time (months) from inclusion to activation of such actions between the intervention group and control group as mean time (months) from inclusion to activation of such actions. This outcome will indicate if DBED may lead to earlier supportive actions for children.
- Effect of DBED on parental stress analyzed as the difference in total scores of the PSS between the intervention group and the control group [12]. Measurements will be recorded every sixth month during the project period. This outcome will indicate if DBED may lead to less or more parental stress and more or less self-confident parents.

Process outcomes:

- The factor structure of EWQ as reported by parents and teacher, respectively, prior to the parent-teacher conference and as reported DBED concluded concern from the parent-teacher conference, primarily at baseline, but also at the following half-year registrations for comparative reasons.
- Agreement between (i) reported concern and DBED concluded concern, respectively, measured by total EWQ score and EWQ sub-scores, and (ii) mental health measured by SDQ Total difficulties score, each sub-scale score, and two SDQ impact scores, respectively, at baseline.
- Agreement between parent’s and teacher’s mental health report measured by agreement between parent and teacher SDQ scores and the above mentioned SDQ impact scores at baseline and after 12, 24, and 36 months, respectively.
- Agreement between parents and teacher in possible concerns for the child measured by percentage of EWQ items rated as the three different response options, respectively (satisfied; a bit uncertain; concerned), at baseline and after 12, 24, and 36 months, respectively).
- Change from reported to concluded concern during the parent-teacher conference measured similarly as the preceding outcome.
- Satisfaction with the DBED after all parent-teacher conferences measured by the study-specific satisfaction questionnaires for parents and teachers, respectively.
- Predictors of mental health change during the 5-year follow-up. Mental health is measured by SDQ-scores (Total difficulties score, sub-scales and impact scores) and the predictors (at baseline) are measured by:
  - ◦ Parent’s educational level
  - ◦ Satisfaction with economic situation
  - ◦ Social support
  - ◦ Single parenting
  - ◦ Close relatives in the vicinity
  - ◦ Norwegian language at home?
  - ◦ Is Norway parent’s country of origin?
  - ◦ Major illness or handicap
  - ◦ Level of parental concern
  - ◦ Level of teacher concern
  - ◦ Level of DBED concluded concern
  - ◦ Parent-teacher agreement after the parent-teacher conversation at baseline
  - ◦ Level of parental stress
- Predictors of parental and teacher concern, respectively. Concern is measured by parent and teacher EWQ total score at baseline and changes throughout the 3-year follow-up period in kindergarten, and the predictors are the same as for mental health change.
- Predictors of parental stress. Parental stress is measured at baseline and changes throughout the total follow-up period of 5 years, and the predictors are the same as for mental health change (except parental stress).
- Predictors of parent satisfaction. Parent satisfaction is measured by the study-specific satisfaction questionnaire at baseline and changes throughout the 3-year follow-up period in kindergarten, and the predictors are the same as for mental health change.

### Participant timeline {13}

**Table:** 

	Study period											
Year^**a**^	0	1			2		3		4		5	
Timepoint (months after start of kindergarten year)	10	3	4–6	10–12	4–6	10–12	4–6	10–12	4–6	10–12	4–6	10–12
Randomization	x											
**Enrolment**												
Informed consent^b^		x										
**Intervention**												
DBED^c^			x	x	x	x	x	x				
TAU^c^			x	x	x	x	x	x				
**Assessment**												
Baseline demographics and health information			x									
EWQ			x	x	x	x	x	x				
Conclusions DBED			x	x	x	x	x	x				
Actions^d^			x	x	x	x	x	x				
SDQ^e^			x	x	x	x	x	x	x	x	x	x
PSS			x	x	x	x	x	x	x	x	x	x
**Process evaluation**^f^												
Parent and teacher satisfaction^g^			x	x	x	x	x	x				
Interviews of parents and kindergarten teachers				x				x				
Audio recordings of parent-teacher conferences				x				x				

^a^Kindergarten year starts July 1^st^

^b^Informed consent is obtained a few weeks before intervention

^c^DBED and TAU are accomplished every 6 months

^d^Specified supportive actions within or outside the kindergarten, differentiated between old and new ones

^e^Data are collected 1 to 3 weeks after the intervention, but the scores relate to the child’s performance during the last 6 months

^f^Several of the questionnaires intended for primary and secondary outcomes, such as EWQ, Conclusions DBED, SDQ, and PSS, are also used in the process evaluation; see the “Outcomes {12}” section

^g^Includes also free text comments related to responder’s experiences of participating in the DBED method

### Sample size {14}

The participating children are expected to represent the general population and are not a clinical sample with a presumed high level of mental symptoms. Hence, even if DBED will have a promoting effect on mental health among the children, the difference between the intervention and the control group will probably be small. In the absence of other similar studies, we chose a difference of 0.8 in SDQ Total Difficulties Scale score based on discussions in the project group. Then, we powered the study to be able to detect such a difference between the implementation and the control group. Mplus was used for Monte Carlo simulations assuming the participation of 20 kindergarten (clusters) with ten participating subjects within each cluster and a baseline SDQ mean 5.5 (English norm data: 5.7) SD = 5.5 (σ^2^_within_ = 30.25). The group differences were represented with a predictor of the slope factor in a latent growth curve model. The model assumed normative stability in the controls (mean change = 0.0) and a reduction of −0.8 in the intervention group during the total period. Group sizes were set to be equal. Some between-cluster variation was specified in the intercept factor (σ^2^_between_ = 2.0) to give expected ICC = 0.10 in the six outcome variables over time. The attrition was assumed to be up to 25%. The number of replications was 1000. To achieve the difference of 0.8 with a sample size of *n* = 100 in each group, the statistical power for the testing of group difference was found to be 95%.

### Recruitment {15}

By collaboration agreements, the three kindergarten organisations through their kindergarten managers have committed to facilitate recruitment of participants. Members of the project group have offered extensive information and training for teachers and parents in all kindergartens (see under the “ Strategies to improve adherence to interventions {11c}” section). A QR code linking to registration for potential participating parents is available in fliers and in the meetings with eligible parents. Consenting parents are offered two cinema tickets. During the recruitment period, the managers of every participating kindergarten are given feedback as to the consent rate in their own kindergarten compared to all the others’. Recruitment will continue every new kindergarten year (August to July) until consent is achieved from a minimum of 100 children in each arm.

## Assignment of interventions: allocation

### Sequence generation {16a}

To prevent contamination from intervention to control cases, the separate kindergartens will be assigned to either intervention or control condition. Because the kindergartens will be recruited from three different organisations, randomizing will be performed within each organisation (block randomisation). The randomisation will be computer-generated by a staff member at Research Unit for Health Surveys, University of Bergen, who is not a part of the PRO-DIALOG project.

### Concealment mechanism {16b}

N/a. To prevent contamination from intervention to control cases, the informed consent to the participants in the control kindergartens will not contain the same detailed information about DBED as the participants in the intervention kindergartens. Hence, intervention and control conditions were assigned at the kindergarten level before the recruitment of parents. To be able to motivate parents to participate, kindergarten teachers in the intervention kindergartens were trained in the DBED method before recruitment. The kindergarten managers were informed about the allocation about 6 months before recruitment.

### Implementation {16c}

An external person who has no other connection to the project will generate the randomization sequence and allocate a study arm to each kindergarten using a pre-fixed list of the kindergartens.

## Assignment of interventions: blinding

### Who will be blinded {17a}

N/a: This is an open-label trial.

### Procedure for unblinding if needed {17b}

N/a. This is an open-label trial.

## Data collection and management

### Plans for assessment and collection of outcomes {18a}

Data from the study will solely be collected by online digital questionnaires completed by teachers and parents via PC, tablet, or smartphone. The questionnaires are built on the EasyTrial platform [13] and will be distributed and collected by the study coordinator at Research Unit for Health Surveys [14]. Questionnaires are distributed by SMS (parents) or work e-mail (teachers) where a personal link to the questionnaire is attached. The answers are directly transferred to the EasyTrial platform.

All kindergarten teachers will be informed about the complementing procedures, and every questionnaire will contain instructions for users. Teachers in the intervention kindergartens are trained in the DBED procedure by a training session involving practical role-play.

The following questionnaires are used:

- Baseline information (demographics, social support, health related information)
  - ◦ Parent’s educational level (values 0–3)
  - ◦ Satisfaction with economic situation (Visual analogue scale, values 0–10)
  - ◦ Social support, adapted from the PIRM study [15] containing nine items. See Additional file 2 for details.
  - ◦ Single parenting (values 0–1)
  - ◦ Close relatives in the vicinity (values 0–1)
  - ◦ Norwegian language at home? (values 0–1)
  - ◦ Is Norway parent’s country of origin? (values 0–1)
  - ◦ Major illness or handicap (child) (values 0–1)
- EWQ (only for the intervention group) [10] is a novel tool for assessing parents and kindergarten teachers’ possible concerns for a child. The psychometric properties of EWQ are not yet established but will be subject to analyses in the present study. We will apply the total EWQ score (values 0–74) and EWQ sub-scores (five sub-scores, values from 0–10 to 0–18). EWQ will be scored in two different situations:
  - ◦ Prior to the parent-teacher conference, completed by both each parent and teacher.
  - ◦ As conclusions after the parent-teacher conference, completed by teacher
- SDQ, 2–4 years version [16] is a standard screening tool for mental health symptoms among young children with well-established validity [16] and psychometric properties [17]. We will apply SDQ Total Difficulties Scale score (values 0–40), each sub-scale score (values 0–10), and two SDQ impact scores (values 0–3 and 0–12, respectively) addressing difficulties in general and in four different areas of daily functioning.
- PSS is an 18-item questionnaire (values 0–72) assessing parents’ feelings about their parenting role, exploring both positive aspects (e.g., emotional benefits, personal development) and negative aspects of parenthood (e.g., demands on resources, feelings of stress) [12].
- User satisfaction with the DBED method (only for the intervention group) measured after all parent-teacher conferences by study-specific questionnaires, see Additional file 2.
  - ◦ Parent questionnaire: five items (values 0–15) and optional free text response
  - ◦ Teacher questionnaire: seven items (values 0–21) and optional free text response
- Possible supportive actions initiated, see Additional file 2.
- How specific questionnaires relate to the primary, secondary, and process outcomes, respectively, is described in the “Outcomes {12}” section. Collection of qualitative data for process outcomes is described in the “Data management {19}” section.

### Plans to promote participant retention and complete follow-up {18b}

Participation in the trial means biannual completion of online questionnaires and attending parent-teacher conferences (intervention kindergartens by DBED, control kindergartens by TAU). Participants in the intervention kindergartens will receive EWQ and invitations to DBED parent-teacher conferences by the study coordinator and kindergarten teacher, respectively, and will be given reminders when necessary. Participants in the control kindergartens will be invited to TAU parent-teacher conferences by their kindergarten teacher. The study manager and study coordinator will have regular contact with every kindergarten manager and participating teacher for continued consciousness and compliance with the study.

### Data management {19}

All data will be entered digitally by the participants themselves through questionnaires in the EasyTrial platform [13]. EasyTrial has built-in data entry validations such as range checks, data type checks, logic checks, and conditional logic checks to promote data quality. Blocks of raw data will periodically be exported by the study coordinator as SPSS files to a secure research server controlled by Helse Vest IKT. To preserve the integrity and validity of the self-reported data, a statistician will organize the data for further analysis (recode variables, compute sum scores, merge different measurements, make long/wide format files to match the analyses, aggregate and disaggregate scores within subjects) and remove missing data. The database will also be exported in full at the end of the study after database lock.

Audio recordings of parent-teacher conferences and subsequent interviews of parents and teachers (see the "Methods for additional analyses{20b}" section) will be collected by a hand-held digital recorder. A researcher will personally collect the recorders in the kindergarten, and speech from the recordings will be transcribed to text using Whisper artificial intelligence software in a PC without an internet connection. All names mentioned in the conversations are automatically changed to another name, and all dialect is changed to the default Norwegian Bokmål language. In this way, it should not be possible for the researcher to recognize who has participated in the conversation.

### Confidentiality {27}

Before consenting to participate in the study, interested parents of eligible children will provide personal contact information through the online questionnaire system. If the parents do not consent within 30 days, this information is deleted automatically. By consent, this information and further personal information of the parents and their child will be stored together with the rest of the collected data. After being removed from the data files, name and national identity number will be linked to unique ID numbers found in the main data files and stored in a separate key file. Only the study manager and study coordinator will have access to the key file. After project closure (year 2040) all data will be stored for five more years for control purposes.

### Plans for collection, laboratory evaluation, and storage of biological specimens for genetic or molecular analysis in this trial/future use {33}

N/a.

## Statistical methods

### Statistical methods for primary and secondary outcomes {20a}

- Primary outcome:
  - ◦ Possible differences between children in intervention and control kindergartens in mental health trajectories will be estimated by Latent growth curve (LGC) models (gives equal results as linear mixed/multilevel models, given similar parameter specification). Both random intercept fixed slope and random intercept random slope will be analyzed, meaning models with no variance in change (slope) and models with individual differences in slope (random effects). Then the full unstructured matrix with random intercept, random slope, and the covariance between intercept and slope will be tested. Initially, linear change over time will be tested. If not supported with data, different non-linear change models will be explored (quadratic, cubic, piecewise growth). Then, substantial predictors will be entered into the model and analyzed. Also, within level residual structures will be tested.
- Secondary outcomes:
  - ◦ Possible differences between children in intervention and control kindergartens in parental stress and time to initiated locally available supportive action, respectively, will be analyzed in a similar way to the primary outcome.
- Process measures:
  - ◦ Factor structure will be estimated by confirmatory factor analysis (CFA).
  - ◦ Agreements between:
    - ▪ Reported concern (EWQ) and DBED concluded concern (EWQ), respectively, and mental health (SDQ): correlations (Pearson’s and Spearman), Cohen’s (weighted) Kappa, CFA: uni-, bi-, and tri-factor models representing different sources of information (parent 1, parent 2, teacher. Latent difference score (LDS) models will also be explored for common levels and differences within and between families.
      - Parent’s and teacher’s mental health report (SDQ): correlations (Pearson’s and Spearman), CFA, including method factors, LDS models.
      - Parents’ and teacher’s possible worries for the child: Cross table with chi-quadrat test, Cohen’s weighted Kappa, CFA, and LDS models.
    - ▪ Change from reported (EWQ) to concluded concern (EWQ) during the parent-teacher conference: cross table with chi-quadrat test, Cohen’s weighted Kappa, CFA/LDS models.
    - ▪ Satisfaction with DBED:
      - Initial: distribution of scores and mean values of each item. Mean value of total score.
      - Follow-up: repeated measures of mean values of each item and total scores and LGC models.
    - ▪ Predictors of mental health change, parent concern, teacher concern, parent satisfaction, parental stress: LGC models with predictors (conditional LGC models).
- Statistical principles
  - ◦ Level of statistical significance: We will use the threshold of *p* = 0.05 for the main analysis. However, the threshold perspective has been debated, and there is considerable literature claiming another perspective as a relevant alternative. This implies the use of exact p-values as a degree of certainty of the generalization from sample to population. In the exploratory part of the project, this practice will be used. In addition, we will calculate effect sizes to evaluate the results beyond statistical significance.
  - ◦ Adjustment for multiplicity: Detailed power analysis (Monte Carlo analysis) was used to decide sample size for satisfactory power. Adjustment is irrelevant for this analysis. Regarding other statistical analyses, analyses may introduce risk for Type 1 errors when testing family-wise tests. When testing specific hypotheses (see above), no adjustment should be necessary. When exploring differences and relations not pre-specified in hypotheses (family wise tests), Bonferroni corrections will be used.
  - ◦ Confidence intervals to be reported: 95%
  - ◦ Methods used for assumptions to be checked for statistical methods: Residuals will be saved and checked for normality and homoscedasticity. Relations will be plotted and linearity in relations evaluated.
  - ◦ Details of alternative methods to be used if distributional assumptions do not hold: Bootstrapping will be used if normality does not hold. However, larger samples are robust for such deviations.
    If survival models (Cox and non-proportional parametric hazard models based on Weibull distribution), the proportional assumption will be tested. In addition, covariates will also be tested as time-dependent covariates to explore whether they relate to events differently over time.

### Interim analyses {21b}

N/a.

### Methods for additional analyses (e.g., subgroup analyses) {20b}

The study includes a qualitative approach with process data collection from audio recordings of parent-teacher conferences and subsequent interviews of parents and teachers, respectively. The audio recordings and the interviews will be transcribed verbatim and analyzed with conversation analysis (CA) [18, 19] and the methodologies of Kvale and Brinkmann [20].

### Methods in analysis to handle protocol non-adherence and any statistical methods to handle missing data {20c}

Non-adherence will be when participants in the intervention group do not complete the parent-teacher conference due to intercurrent obstacles or drop-out. All analyses will be handled as intention to treat. Missing data will be handled by multiple imputation.

### Plans to give access to the full protocol, participant-level data, and statistical code {31c}

The full protocol will be available on request. Likewise, completely de-identified data and statistical code will be available post-trial after the results are published.

## Oversight and monitoring

### Composition of the coordinating centre and trial steering committee {5d}

The principal investigator (IB) has the day-to-day responsibility for leading and coordinating all activities and collaborators in the project. The Research unit for health surveys (RUHS) at University of Bergen issues and receives all questionnaires from parents and teachers and transfers the data to a secure research server. Every second week there is a project meeting with the PI, two researchers (GJW and M-BP), two representatives from RUHS and a PhD candidate (VKL). Twice a year there is a meeting in the extended project group supplied by two user representatives (one parent and one kindergarten teacher), a statistician (RG) and contact persons from Øygarden municipality and Eventus kindergarten, respectively. Biannually, there is also a meeting with the international collaborators (CG, PW, CM, AVJ). Finally, a stakeholder meeting with representatives from the different kindergarten owners and University of Bergen and Western Norway University of Applied Sciences is held twice a year. The project itself is owned by Haukeland University Hospital (HUS) and located in the research unit of Division of Mental Health Services. The project is monitored by the Research and Education Department at HUS.

### Composition of the data monitoring committee, its role and reporting structure {21a}

In this low-risk study, a data monitoring committee was not considered necessary.

### Adverse event reporting and harms {22}

The risk of potential harm from the intervention, the prepared and structured parent-teacher conference, is regarded as negligible. Interpersonal challenges may of course arise in any parent-teacher conference and will be managed locally in individual kindergartens.

### Frequency and plans for auditing trial conduct {23}

No auditing is planned. However, on request, the relevant authorized body^1^ can audit at any stage of the research project.

### Plans for communicating important protocol amendments to relevant parties (e.g., trial participants, ethical committees) {25}

Important protocol modifications will be approved by the Regional Committee for Medical and Health Research Ethics and will be reported to all collaborating organizations and participating kindergartens.

### Dissemination plans {31a}

Results from the study will be presented at local and national conferences aimed at professionals and administrators of health and educational services, together with politicians and researchers interested in the promotion of children’s mental health. Likewise, results will be presented at national and international scientific conferences in the field of mental health, education, and health promotion. Our findings will be submitted to peer-reviewed international journals in the fields of mental health, education, and health promotion. Finally, the educational institution for kindergarten teachers in Bergen (Western Norway University of Applied Science) would, as a partner in the PRO-DIALOG project, be a natural starting point for curriculum development based on results and experiences from the project.

## Discussion

In societies with high preschool uptake, such as Norway, kindergartens have great potential for mental health promotion in young children. Targeted interventions in preschools, where children may require additional support, have been proven to deliver far greater societal returns compared to interventions introduced later in life [4]. We believe that the PRO-DIALOG project holds the potential to generate such an impact and have both short-term and long-term benefits for individuals and society.

To our knowledge, this is the first trial to investigate a systematic parent-teacher conference in kindergartens aiming to promote child mental health. To avoid contamination of the intervention on control cases, a block randomized design was regarded as appropriate. With this design, all teachers to the children of the eligible birth cohorts in the intervention kindergarten receive training in DBED. Likewise, all the eligible parents are informed and invited to participate.

Despite the RCT design, assuming equality between the intervention and control group might be optimistic. However, adjusting for differences by both a multi-variable and multi-level approach will mitigate such a bias.

As far as we know, there is no convention for what a significant difference in mental health level (SDQ Total difficulties score after 3 years) is in a community-based population exposed to a secondary preventive measure. Hence, our power estimation is based on a somewhat uncertain assumption. The recruitment of parents will go on until the minimum number of participants is reached; however, it will not stop before all invited parents have been given the opportunity to consent to participation. So, the number of participants will probably exceed 100 + 100.

Along with the core trial, aspects beyond the long-term outcome of child mental health are addressed, such as screening properties and social validity of this novel method. For further studies of DBED, these process data will be of great interest.

### Trial status

Protocol version: Third version, November 2024.

Recruitment started January 2024 and will be completed during January 2025.

## Supplementary Information

Additional file 1: A logic model of Pro-Dialog.

Additional file 2: Various questionnaires in the PRO-DIALOG study.

## Data Availability

Only the principal investigator (IB) will have access to the final data.

## Abbreviations

PRO-DIALOG: Promoting mental health in young children—a dialogue-based approach in kindergartens
DBED: Dialogue-Based Early Detection
EWQ: Early Worry Questionnaire
SDQ: Strengths and Difficulties Questionnaire
PSS: Parental Stress Scale
CAMHS: Child and Adolescent Mental Health Service

## Footnotes

1 Such as the Regional Committee for Medical and Health Research Ethics, the Norwegian Data Protection Authority, or the Norwegian Board of Health Supervision.
