# Supplementary material for: PRO-DIALOG—the effect of a novel dialogue-based parent-teacher conference on mental health in kindergarten children: a cluster randomized controlled trial
Source: Trials. 2025 Aug 21;26:299. doi: 10.1186/s13063-025-08980-x (PMC12372322; doi:10.1186/s13063-025-08980-x)
Supplement: Supplementary file 1 — Additional file 1: A logic model of Pro-Dialog. [file 13063_2025_8980_MOESM1_ESM.pptx]

## Slide 1
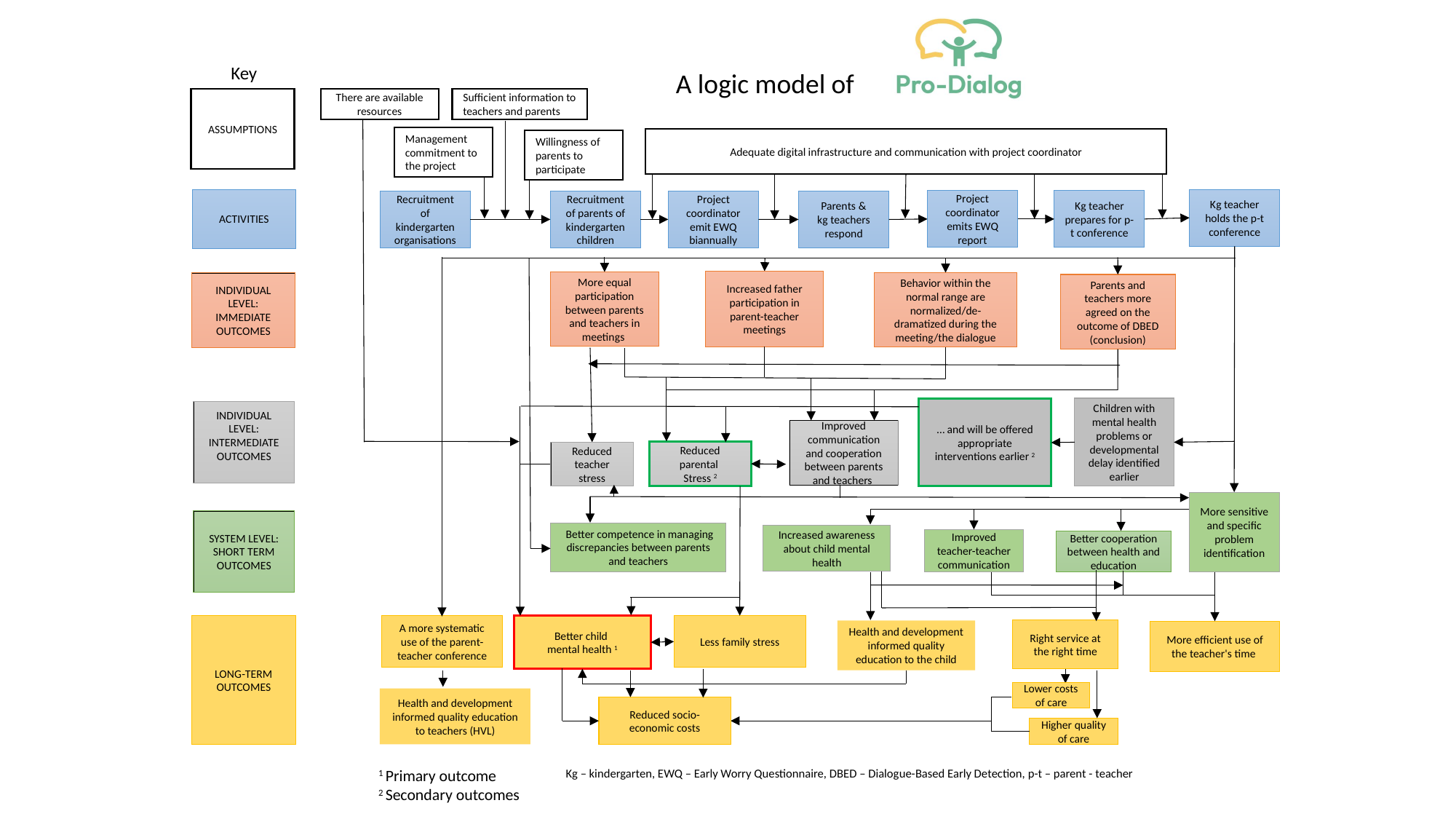

Key
A logic model of
There are available resources
Sufficient information to teachers and parents
ASSUMPTIONS
Management commitment to the project
Adequate digital infrastructure and communication with project coordinator
Willingness of parents to participate
ACTIVITIES
Kg teacher holds the p-t conference
Project coordinator emits EWQ report
Kg teacher prepares for p-t conference
Recruitment of kindergarten organisations
Recruitment of parents of kindergarten children
Project coordinator emit EWQ biannually
Parents &
kg teachers respond
Increased father participation in parent-teacher meetings
More equal participation between parents and teachers in meetings
Behavior within the normal range are normalized/de-dramatized during the meeting/the dialogue
INDIVIDUAL LEVEL: IMMEDIATE OUTCOMES
Parents and teachers more agreed on the outcome of DBED (conclusion)
Children with mental health problems or developmental delay identified earlier
… and will be offered appropriate interventions earlier 2
INDIVIDUAL LEVEL: INTERMEDIATE OUTCOMES
Improved communication and cooperation between parents and teachers
Reduced parental
Stress 2
Reduced teacher stress
More sensitive and specific problem identification
SYSTEM LEVEL: SHORT TERM OUTCOMES
 Better competence in managing discrepancies between parents and teachers
Increased awareness about child mental health
Improved teacher-teacher communication
Better cooperation between health and education
LONG-TERM OUTCOMES
A more systematic use of the parent-teacher conference
Better child
mental health 1
Less family stress
Right service at the right time
Health and development informed quality education to the child
More efficient use of the teacher's time
Lower costs of care
Health and development informed quality education to teachers (HVL)
Reduced socio-economic costs
Higher quality of care
1 Primary outcome
2 Secondary outcomes
Kg – kindergarten, EWQ – Early Worry Questionnaire, DBED – Dialogue-Based Early Detection, p-t – parent - teacher
