# Supplementary material for: PRO-DIALOG—the effect of a novel dialogue-based parent-teacher conference on mental health in kindergarten children: a cluster randomized controlled trial
Source: Trials. 2025 Aug 21;26:299. doi: 10.1186/s13063-025-08980-x (PMC12372322; doi:10.1186/s13063-025-08980-x)
Supplement: Supplementary file 2 — Additional file 2: Various questionnaires in the PRO-DIALOG study. [file 13063_2025_8980_MOESM2_ESM.docx]

Various questionnaires in the PRO-DIALOG study

1. **Social support questionnaire, adapted from the PIRM study** (NORCE 2024)

|  | **Fits completely = 2** | **Fits partially = 1** | **Does not fit = 0** |  |
| --- | --- | --- | --- | --- |
| I feel close to my family |  |  |  |  |
| I feel close to my friends |  |  |  |  |

| **All in all, do you think you have sufficient** | **Yes, to a large extent = 2** | **Yes,  to some extent = 1** | **No,  to a small extent = 0** |
| --- | --- | --- | --- |
| **Contact with others** |  |  |  |
| Care / support |  |  |  |
| Understanding / respect |  |  |  |
| Other practical help |  |  |  |

|  | **No, none = 0** | **I have 1 confidant =1** | **I have 2 confidants = 2** | **I have several confidants = 3** |
| --- | --- | --- | --- | --- |
| Do you have any confidants (other than your spouse/partner) who you can talk to about most things? |  |  |  |  |

|  | **No, none  = 0** | **Yes, 1 to 2 people = 1** | **Yes, more than 2 people  = 2** |
| --- | --- | --- | --- |
| Do you have someone besides your spouse/partner who you can seek advice from in difficult situations? |  |  |  |

|  | **No, none  = 0** | **Yes, 1 = 1** | **Yes, 2 = 2** | **Yes, 3-4 = 3** | **Yes, 5 or more = 4** |
| --- | --- | --- | --- | --- | --- |
| Do you have any confidants (other than your spouse/partner) who you can talk to about most things? |  |  |  |  |  |

NORCE. Parenting Interventions for Families with Refugee Background (PIRM) 2024 [Available from: https://www.norceresearch.no/en/projects/parenting-interventions-for641 families-with-refugee-background-pirm.

1. **User-satisfaction questionnaires**

|  | **For parents** | **No,** | | **Yes,** | | **Not relevant** |
| --- | --- | --- | --- | --- | --- | --- |
|  |  | **not at all** | **not much** | **quite a bit** | **very much** |  |
| 1 | Was it difficult to understand why I/we should complete the questionnaire? | **= 3** | **= 2** | **= 1** | **= 0** |  |
| 2 | Was it difficult to complete the questionnaire? | **= 3** | **= 2** | **= 1** | **= 0** |  |
| 3 | Was it a good experience to talk with the educational supervisor during the review of the questionnaire in the meeting? | **= 0** | **= 1** | **= 2** | **= 3** |  |
| 4 | Do you think the questionnaire was useful? | **= 0** | **= 1** | **= 2** | **= 3** |  |
| 5 | Do you think the meeting was useful? | **= 0** | **= 1** | **= 2** | **= 3** |  |

|  | **For teachers** | **No,** | | **Yes,** | | **Not relevant** |
| --- | --- | --- | --- | --- | --- | --- |
|  |  | **not at all** | **not much** | **quite a bit** | **very much** |  |
| 1 | Was it difficult to complete the questionnaire? | **= 3** | **= 2** | **= 1** | **= 0** |  |
| 2 | Was it difficult to introduce the questionnaire in the parent-teacher conferences? | **= 3** | **= 2** | **= 1** | **= 0** |  |
| 3 | Was the questionnaire an obstacle to a good parent-teacher conference? | **= 3** | **= 2** | **= 1** | **= 0** |  |
| 4 | Was the method helpful in clarifying, together with the parents, whether there was a concern or not for the child? | **= 0** | **= 1** | **= 2** | **= 3** |  |
| 5 | Was it difficult to summarize what were the concerns for the child? | **= 3** | **=2** | **= 1** | **= 0** |  |
| 6 | Did the method make it easier, together with the parents, to come up with possible measures for the child? | **= 0** | **= 1** | **= 2** | **= 3** |  |
| 7 | Did the method contribute to a good dialogue with the parents about any concerns for the child? | **= 0** | **= 1** | **= 2** | **= 3** |  |

1. **Possible supportive actions initiated**

| **Within the kindergarten** |  |
| --- | --- |
| Systematic observation |  |
| Structured facilitation of the environment |  |
| Individual follow-up of the child |  |
| Primary contact by adult |  |
| Play-/friend-group |  |
| Language group |  |
| Child conversation |  |
| Guidance for parents |  |
| Advice from a special education teacher |  |
| Follow-up of a special education teacher at a low-threshold level |  |
| Others |  |

| **Outside the kindergarten** |  |
| --- | --- |
| Contact meeting with the educational and psychological counselling service |  |
| Consultation from the educational and psychological counselling service |  |
| Interdisciplinary discussion meeting |  |
| Assessment at the educational and psychological counselling service |  |
| Speech therapist |  |
| Child health clinic |  |
| Physiotherapist |  |
| Occupational therapist |  |
| Family doctor |  |
| Child welfare |  |
| Mental health service for children and families at the municipal level |  |
| Anti-bullying measures at the municipal level |  |
| Child and adolescent mental health service |  |
| Pediatric hospital |  |
| Others |  |
